# Rasagiline Ameliorates Olfactory Deficits in an Alpha-Synuclein Mouse Model of Parkinson's Disease

**DOI:** 10.1371/journal.pone.0060691

**Published:** 2013-04-03

**Authors:** Géraldine H. Petit, Elijahu Berkovich, Mark Hickery, Pekka Kallunki, Karina Fog, Cheryl Fitzer-Attas, Patrik Brundin

**Affiliations:** 1 Neuronal Survival Unit, Wallenberg Neuroscience Center, Department of Experimental Medical Science, BMC B11, Lund University, Lund, Sweden; 2 Teva Pharmaceutical Industries Ltd., Global Innovative Products, Petach-Tikva, Israel; 3 H. Lundbeck A/S, Neurology, Copenhagen, Denmark; 4 H. Lundbeck A/S, Neurodegeneration-1, Copenhagen, Denmark; 5 Van Andel Research Institute, Center for Neurodegenerative Science, Grand Rapids, Michigan, United States of America; University of Florida, United States of America

## Abstract

Impaired olfaction is an early pre-motor symptom of Parkinson's disease. The neuropathology underlying olfactory dysfunction in Parkinson's disease is unknown, however α-synuclein accumulation/aggregation and altered neurogenesis might play a role. We characterized olfactory deficits in a transgenic mouse model of Parkinson's disease expressing human wild-type α-synuclein under the control of the mouse α-synuclein promoter. Preliminary clinical observations suggest that rasagiline, a monoamine oxidase-B inhibitor, improves olfaction in Parkinson's disease. We therefore examined whether rasagiline ameliorates olfactory deficits in this Parkinson's disease model and investigated the role of olfactory bulb neurogenesis. α-Synuclein mice were progressively impaired in their ability to detect odors, to discriminate between odors, and exhibited alterations in short-term olfactory memory. Rasagiline treatment rescued odor detection and odor discrimination abilities. However, rasagiline did not affect short-term olfactory memory. Finally, olfactory changes were not coupled to alterations in olfactory bulb neurogenesis. We conclude that rasagiline reverses select olfactory deficits in a transgenic mouse model of Parkinson's disease. The findings correlate with preliminary clinical observations suggesting that rasagiline ameliorates olfactory deficits in Parkinson's disease.

## Introduction

Parkinson's disease (PD) patients not only exhibit motor dysfunction, but also multiple non-motor symptoms [1]. Hyposmia, i.e. impaired detection, discrimination and/or identification of odors affects 70–6% of PD patients [2], [3], typically several years before onset of motor symptoms [4]. Hyposmia might be an useful sign when predicting who will develop PD later [5].

The causes of olfactory impairments in PD are not understood. In PD, Lewy bodies and Lewy neurites are present in mitral cells and in the inner plexiform layer of the olfactory bulb (OB), and in cells along the olfactory neural pathways [6]. Braak et al. (2003) have suggested that these α-synuclein aggregates appear before the onset of motor symptoms.

Either these protein aggregates or changes in OB neurogenesis might contribute to olfactory deficits in PD. The numbers of proliferating cells in the subventricular zone and neural precursors in the OB are reduced [7], and some animal PD models exhibit OB neurogenesis changes [8], [9].

Rasagiline (N-propargyl-1-(R)-aminoindan) is an irreversible monoamine oxidase (MAO)-B inhibitor, prescribed as monotherapy in early-stage PD and as an adjunct to levodopa in moderate to advanced PD [10]. It reduces motor deficits and ameliorates motor fluctuations [11]–[13]. A double–blind, delayed-start trial (ADAGIO) indicated that early rasagiline treatment provides benefits consistent with a possible disease-modifying effect [14]. Rasagiline is reported to be neuroprotective in different animal models of neurodegeneration [15]–[17]. Interestingly, preliminary evidence suggests that rasagiline improves olfaction in PD [18], [19] and ongoing clinical studies address this possibility [NCT00902941, NCT01007630].

To investigate the effect of an accumulation of wild-type α-synuclein, we studied a transgenic mouse model of PD expressing human wild-type α-synuclein under the control of the mouse α-synuclein promoter, which is likely to lead to an expression pattern of the human α-synuclein that is similar to the pattern of endogenous mouse α-synuclein expression. We first characterized olfactory deficits in a transgenic mouse model of PD expressing human wild-type α-synuclein. Subsequently, we monitored the effects of rasagiline on these deficits and OB neurogenesis.

## Materials and Methods

### Ethics Statement

This study was carried out in strict accordance with the recommendations in the Guide for the Care and Use of Laboratory Animals of the National Institutes of Health. The Malmö-Lund Animal Ethical Committee approved all experimental procedures (permit number: M55-09). All efforts were made to minimize suffering.

### Transgenic mice and rasagiline treatment

We studied 3, 10–11 and 18 month-old F28 α-synuclein mice (α-syn mice, provided by H. Lundbeck A/S, Denmark) overexpressing wild-type human α-synuclein under the control of the partial mouse α-synuclein promoter [20]. Western blot analysis showed that the level of α-synuclein in total brain lysates of the α-syn mice is slightly less than 3 fold compared to the wild-type level (data not shown). Moreover, real-time PCR quantification showed an increase of α-synuclein in the striatum of approximately 3 fold in α-syn mice compared to wild-type [20]. Previous immunostaining of human α-synuclein protein indicated a cytoplasmic accumulation of α-synuclein in cell bodies of the hippocampal CA1 region, striatum, thalamus, amygdala and in several cortical layers [21].

The 10–11 month-old mice received either rasagiline in the drinking water (3 mg/kg) or normal drinking water. The liquid intake was carefully monitored for each mouse before the experiment and then every week during the experiment. We did not see any difference in water consumption between wild-type and α-synuclein mice (wild-type: 3.23±0.08 ml/day; α-synuclein mice 3.08 ± 0.08 ml/day; Unpaired t-test p = 0.192). The weight of both wild-type and α-synuclein mice was also similar (wild-type mice: 36.1±0.8 g; α-synuclein mice: 35.6±0.7 g; unpaired t-test p = 0.615). Rasagiline concentration was individually adapted throughout the treatment period according to the water intake and weight for each mouse. The drinking water was changed twice a week. During rasagiline treatment and behavioral testing, animals were kept in individual cages (12 h light/dark cycle), with access to food and water *ad libitum*.

### Behavioral tests

**Figure 1:**
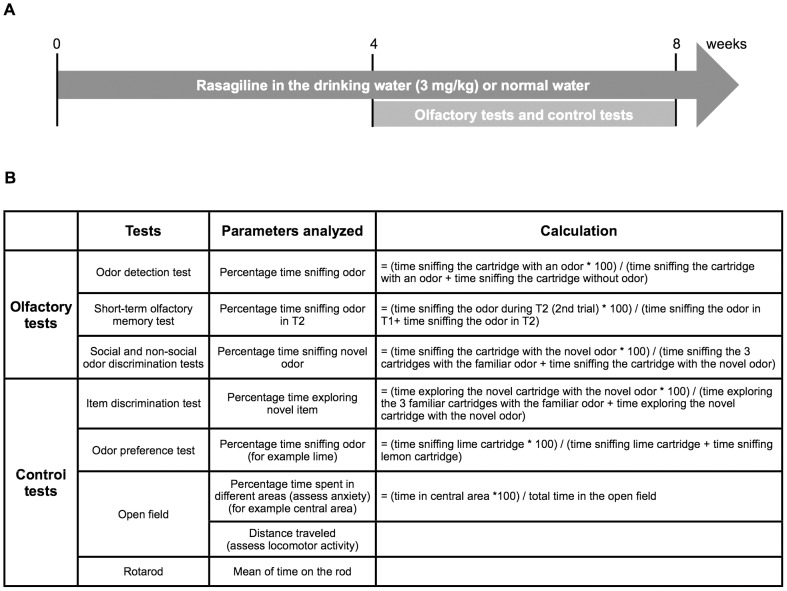
Behavioral experiments: design and parameters analyzed. **A.** Experimental design of the behavioral study. **B**. Olfactory- and control tests and the parameters analyzed from these experiments.

The experimental design of the behavioral study and parameters analyzed for these experiments are described in Figure 1A-B. We began treating mice with rasagiline 4 weeks prior to behavioral testing and continued treatment throughout the period of behavioral testing. To investigate different aspects of olfactory function, we performed a set of olfactory tests (odor detection test, short-term olfactory memory test, social and non-social odor discrimination tests, odor preference test) and control tests (item discrimination test, open field, rotarod) on mice aged 10–11 months. We investigated 21 non-treated wild-type (WT), 18 rasagiline-treated WT, 19 non-treated α-syn mice and 20 rasagiline-treated α-syn mice. The item discrimination, open field, short-term olfactory memory and odor detection tests were performed on only 10 non-treated WT, 9 treated WT, 10 non-treated α-syn mice and 9 treated α-syn mice. In order to assess the progression of olfactory deficits with age we investigated 14 wild-type and α-syn mice aged 3 months, 10 WT and α-syn mice aged 11 months and 14 WT and α-syn mice aged 18 months in the odor detection test, short-term olfactory memory test and the non-social odor discrimination test. All experiments were performed blinded to group identity. Olfactory tests were performed in the mouse's home cage. Two days before commencing the first olfactory test that required the use of plastic cartridges, we placed four cartridges without specific odor in the mouse cage allowing the mice to habituate to the object. Cages were cleared of cartridges or wood blocks and nests 2 h before testing to allow mice to habituate to the experimental environment. The non-social odors used in the olfactory tests (vanilla, lemon, lime, cinnamon, black pepper and anise) were prepared from pure essential oil (Aroma Creative AB, Sweden). Odors were diluted from the pure essential oil stock to the following concentrations 1∶10^8^, 1∶10^6^ and 1∶10^4^. As a social odor, we used wood blocks impregnated with mouse odors for either 7 days (high odor intensity) or 2 days (low odor intensity). The wood blocks were placed in clean cages of individually housed mice and beddings were not changed during the time of odor impregnation (Figure S1 for more details about cartridges and wood blocks). During the olfactory tests, we measured the time spent sniffing cartridges or wood blocks when the following criteria were fulfilled: The nose of the mouse was oriented towards the object and the mouse moved its nose/whiskers (as observed when sniffing). Physical contact with the object was not necessary, however the nose of the mouse had to be close to the object (about max 2 cm away the object). Measurements were made during the tests but all tests were videotaped in order to be able to verify measurements if necessary.

#### Odor detection test

**Figure 2:**
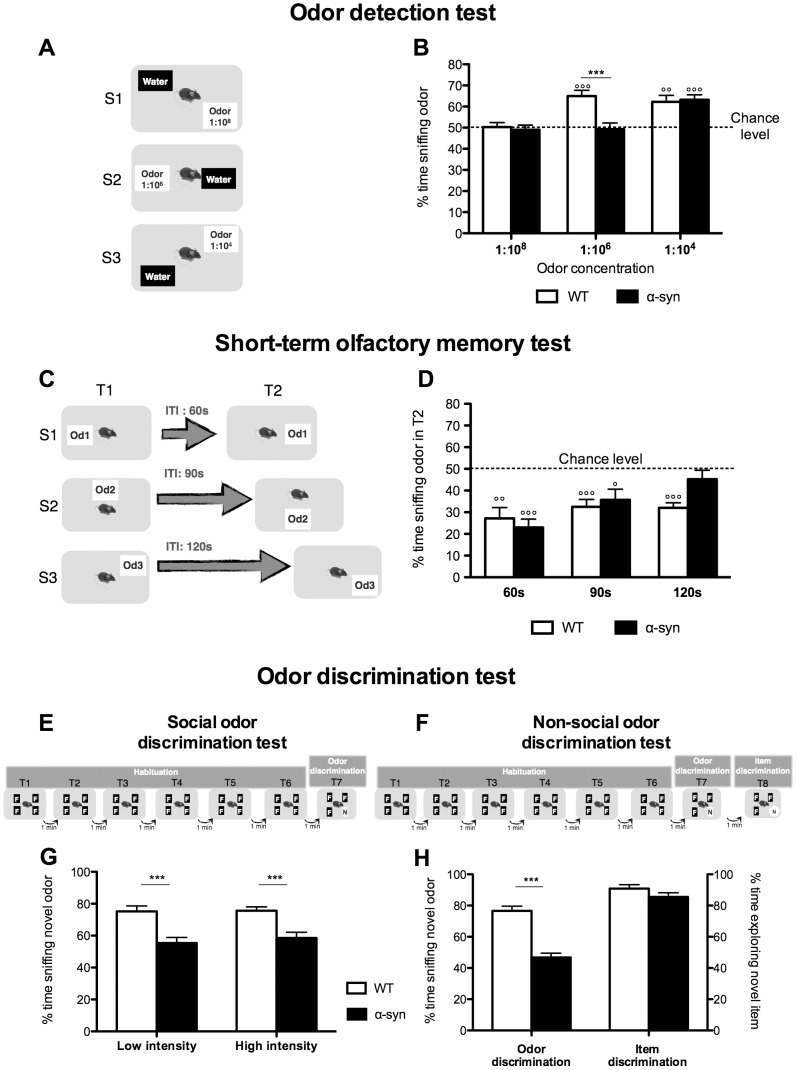
Olfactory deficits in the α-syn mouse model of PD. **A-B:** **Odor detection test. A.** Description of the protocol composed of 3 sessions (S). In each 5-min session, mice were exposed to 2 cartridges, one filled with water, the other with increasing odor concentrations from 1∶10^8^ to 1∶10^4^. **B.** Percentage of time sniffing the odor for the different concentrations. WT mice start detecting the odor at the concentration 1∶10^6^ when percentage of time sniffing the odor is significantly different from the chance level (50%, where mice spent same time sniffing water and odor cartridges) (°°°p<0.001, °°p<0.01, one-sample t-test). α-Syn mice can detect the odor only at 1∶10^4^ (°°°p<0.001, one-sample t-test). At the concentration 1∶10^6^, α-syn mice are significantly impaired compared to WT. N = 10 for each group aged 10–11 months. Statistics: One-sample t-test to compare each value to chance level (50%), (°°°p<0.001, °°p<0.01). Two-way RM ANOVA: odor concentration, p = 0.0001, F(2,36) = 11.96; genotype, p = 0.015, F(1,36) = 7.2; odor concentration×genotype, p = 0.0073, F(2,36) = 5.65; Bonferroni post-hoc (***p<0.001). **C-D: Short-term olfactory memory test. C.** Description of the protocol composed of 3 sessions (S). Each session consisted of two 5 min-trials (T) where mice are exposed to a novel odor separated by an increasing inter-trial time (ITI) from 60 s to 120 s. **D.** Percentage of time sniffing the odor during T2 compared to the total time spent sniffing during both trials. WT mice remember the odor during the 2^nd^ exposure for the 3 ITI tested and their percentage of time sniffing the odor during T2 was significantly different from the chance level (50%, where mice spent same time sniffing the odor during T1 and T2) (°°p<0.01, °°°p<0.001, one-sample t-test). By contrast, short-term olfactory memory of α-syn mice was impaired from an ITI of 120 s (p>0.05, one-sample t-test). However, it was significantly different from chance level at 60 s and 90 s (°°°p<0.001 and °p<0.05 respectively, one-sample t-tests). N = 10 for each group aged 10–11 months. Statistics: One-sample t-test to compare each value to chance level (50%), (°°°p<0.001, °°p<0.01, °p<0.05). **E-H: Odor discrimination test. E and G:** Social odor discrimination test. **E.** Description of the protocol composed of 6 habituation trials where mice are exposed to a familiar odor (F, odor of the tested mouse); and one odor discrimination trial, where one familiar odor is replaced by a novel odor (N, another mouse's odor). This test was performed with low or high odor intensities (wood blocks impregnated with mouse's odor for 2 or 7 days respectively). Each trial lasted 2 min and was separated by 1 min. **G.** Percentage of time sniffing novel odor. For both low and high odor intensities, α-syn mice have impaired odor discrimination with the percentage of time sniffing the odor significantly lower than WT. N = 19–21 for each group aged 10–11 months. Statistics: Two-way RM ANOVA: odor intensity, p = 0.55, F(1,38) = 0.37; genotype, p<0.0001, F(1,38) = 27.1; odor intensity×genotype, p = 0.63, F(1,38) = 0.23; Bonferroni post-hoc (***p<0.001). **F and H:** Non-social odor discrimination test. **F.** Description of the protocol based on the same principle of the social odor discrimination test but using non-social odors (lemon and lime). In the 8^th^ 2 min-trial, an item discrimination trial was added where the usual cartridge, with the novel odor (lime), was replaced by a novel item (a novel type of cartridge associated with the same novel odor, lime). **H.** Percentage of time sniffing the novel odor during the odor discrimination trial and percentage of time exploring the novel item in the item discrimination trial. α-syn mice had significantly impaired odor discrimination of the social odor. By contrast, the ability to discriminate the novel item was similar between WT and α-syn mice suggesting that the discrimination deficit is specific to olfaction. Statistics: unpaired t-test, non-social odor discrimination p<0.0001, N = 19–21 for each group aged 10–11 months; item discrimination p = 0.16, N = 10 for each group aged 10-11 months (***p<0.001).

To determine the threshold of odor detection, we adapted an odor detection test from Breton-Provencher et al. (2009). The test was composed of two or three sessions. In each 5-min session, mice were exposed to two cartridges, one filled with water, the other with increasing novel odor concentrations (vanilla, concentration: 1∶10^8^, 1∶10^6^ or 1∶10^4^, Figure 2A). Normal mice instinctively spend more time sniffing new odors. The test determines if animals can detect the novel odor by comparing the time they spend sniffing the two cartridges.

#### Short-term olfactory memory test

We assessed short-term olfactory memory according to Breton-Provencher et al. (2009). During each of the two or three sessions (S), we exposed mice to a novel odor for two 5 min-trials (T1 and T2) separated by 60, 90 or 120 s inter-trial intervals (ITI) (Figure 2C). The novel odors we used for the ITIs of 60, 90 and 120 s were cinnamon, anise and black pepper (concentration on 1∶10^4^) respectively. If mice remembered the odor from the first trial, they were expected to spend less time sniffing it during the second.

#### Social odor discrimination test

We assessed the ability of the mice to discriminate between social odors and we used two levels of odor intensities (high or low) [22], [23]. First, mice were subjected to six 2-min habituation trials (separated by 1 min ITI) when they were exposed to four wood blocks with a “familiar social odor”. Thereafter, during in the odor discrimination trial, they had to detect that one wood block had been replaced by a block impregnated with a “novel social odor” (Figure 2E). Mice that were able to discriminate between the familiar and novel odors spent more time sniffing the novel odor.

#### Non-social odor discrimination test

The non-social odor discrimination test was identical to the social odor discrimination test with the exception that we used cartridges filled with non-social odors (lemon or lime) instead of wooden blocks (Figure 2F).

#### Item discrimination test

One min after the non-social discrimination test, we performed an 8^th^ 2-min trial aimed at determining whether the mice could discriminate a novel item. Thus, we replaced the cartridge with the novel (lime) odor with a different cartridge type ("novel item") that contained the same lime odor (Figure 2F). We assessed item discrimination by noting an increase in time exploring the novel item. Exploration time includes all different types of exploratory behaviors and interactions with the object. This includes sniffing time as described previously but also time spent touching, manipulating, moving, turning around, or biting the object.

#### Odor preference test

**Figure 3:**
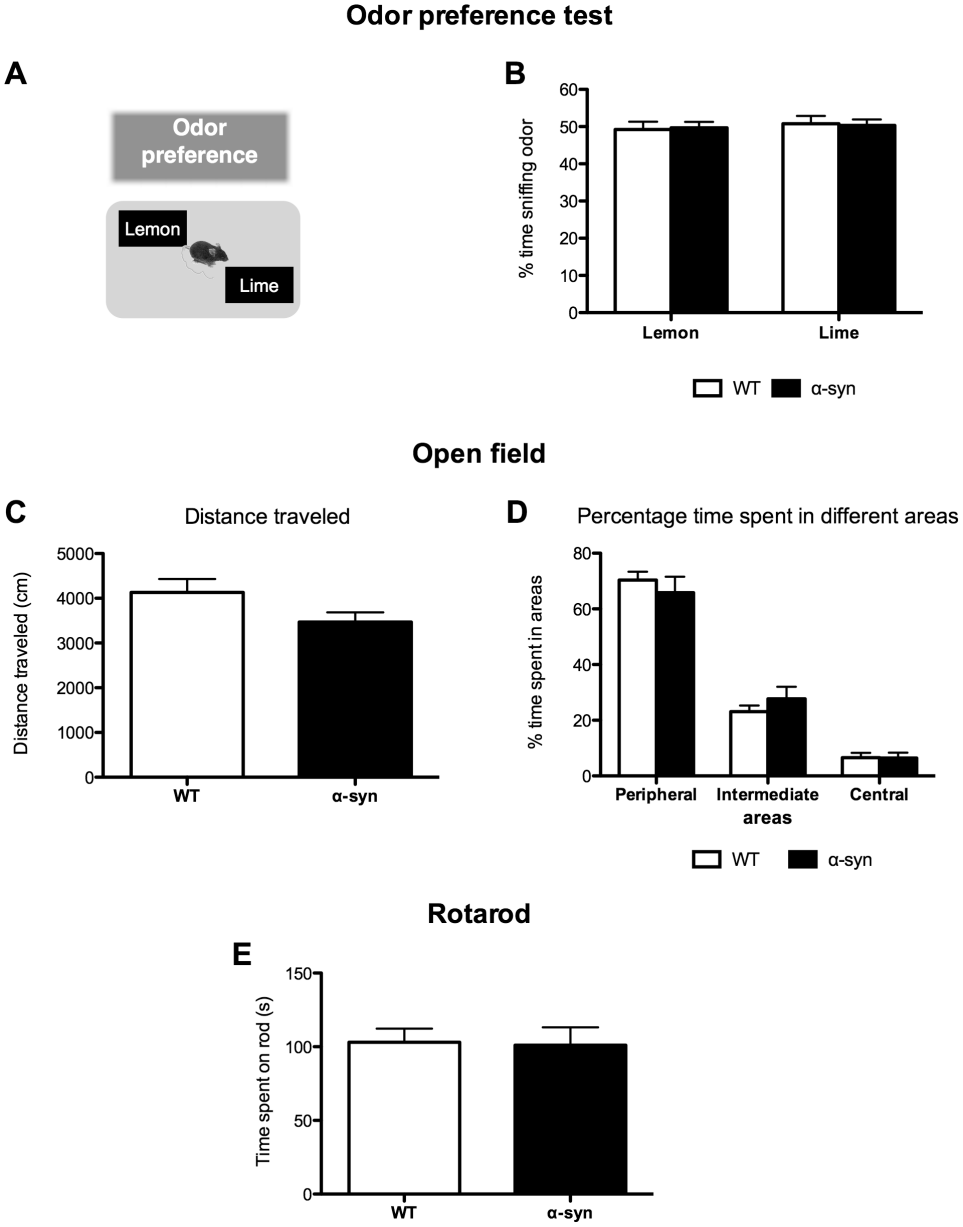
Specificity of the olfactory deficits in α-syn mice. **A**–**B: Odor preference test. A.** Description of the protocol. Two cartridges, filled with either lemon or lime are placed in the cage for 5 min. If mice do not have any odor preference they spend a similar time sniffing either cartridge. **B.** Percentage of time sniffing lemon and lime showing no difference between control and α-syn mice and no difference between lime and lemon odors. N = 19–21 for each group aged 10–11 months. Statistics: two-way ANOVA, odor, p = 0.57, F(1,76) = 0.33; genotype, p = 1.00, F(1,76)≈0; odors×genotype, p = 0.80, F(1,76) = 0.06). **C-D Open field test. C.** Distance traveled in the open field. No significant difference was observed between WT and α-syn mice. α-syn mice show similar locomotor activity to control mice. N = 10 for each group aged 10–11 months. Statistics: unpaired t-test, p = 0.088. **D.** Percentage of time spent in different areas. No significant difference between WT and α-syn mice (p>0.05, two-way ANOVA), suggesting each type of mouse exhibited the same level of anxiety. N = 10 for each group aged 10–11 months. Statistics: two-way ANOVA, genotype, p = 1, F(1,54) = 0; areas, p<0.0001, F(1,54) = 165.1; genotype×areas, p = 0.42, F(2,54) = 0.87; Bonferroni post-hoc between WT and α-syn mice, p>0.05). **E**. **Rotarod test**. Time spent on the rod was similar between both groups of mice. N = 10 for each group aged 10–11 months. Statistics: unpaired t-test, p = 0.9.

The test consisted of a single 5 min-trial during which we exposed mice to two cartridges (one with a lemon and one with a lime odor) and monitored how much time they spent sniffing each odor (Figure 3A). If mice preferred an odor, they spent more time sniffing it.

#### Open field test

Using a video tracking system, we monitored general activity and anxiety status in an open field (42 cm^2^, Noldus, Ethovision, Holland) for 10 min. To assess locomotor activity, we recorded distance traveled. We evaluated anxiety levels based on the time spent in the peripheral, intermediate and central areas [24].

#### Accelerating rotarod test

We assessed motor ability using a rotarod (Rotamex 4/8, Columbus Instruments, USA; 3.8 cm in rod diameter, 4.5 cm in wide section). After a training phase, during which mice had to stay on the rod for 30 s while it was turning at a constant speed (5 rotations per min (rpm)), we tested mice in 4 trials during which the speed of the rotation increased gradually from 4 to 40 rpm over a 5 min period. We averaged the time spent on the rotarod for the four trials.

### BrdU (5-bromo-2'-deoxyuridine) experiment

We studied neurogenesis in four independent groups of mice (6 non-treated WT; 4 rasagiline-treated WT, 4 non-treated α-syn, 4 rasagiline-treated α-syn) that had not undergone behavioral testing. At 10 months of age, we gave them rasagiline in the drinking water for seven weeks. Four weeks prior to sacrificing, we injected them with BrdU (80 mg/kg, i.p., in PBS, pH 7.4) twice daily for 6 consecutive days.

### Histological analysis

#### Immunohistochemistry

We perfused the 12 month-old mice transcardially with 0.9% saline followed by 4% paraformaldehyde and prepared 40 µm thick coronal sections for immunohistochemistry. Free-floating sections were treated with 10% H_2_O_2_ in PBS for 20 min. Specifically for BrdU staining, we treated sections with 2 N HCl in water for 30 min at 37 °C. We used the following primary antibodies: mouse anti-human α-synuclein (1∶2000, Ab36615, Abcam), rat anti-BrdU (1∶100, Oxford Biotechnology OBT0030) and/or mouse anti-NeuN (neuronal nuclei, 1∶100, AB MAB377, Millipore). For detection of human α-synuclein and BrdU antibodies with the chromogen 3,3′diaminobenzidine (DAB), sections were incubated in biotinlylated horse anti-mouse (1∶200, BA-2001, Vector Laboratories) or rabbit anti-rat secondary antibodies (1∶200, E0468, Dako) respectively and then processed using a standard peroxidase-based method (Vectastain ABC kit and DAB kit; Vector Laboratories). For immunofluorescence staining, we used Alexa 488 anti-mouse and Alexa 568 anti-rat secondary antibodies respectively (raised in goat, 1∶200, A11029 or A11077, Invitrogen). Specimen analyses were performed either with a conventional light microscope (Eclipse 80i microscope; Nikon), a confocal laser microscope (Leica TCS SL) or with a stereological setting (Olympus BX50 microscope with a Marzhauser X–Y–Z step motor stage and the Visiopharm Integrator System software, Visiopharm A/S).

#### Cell Counting

We counted the number of BrdU positive cells in the granule cell layer of the OB using a systematic, random counting procedure optical dissector (section interval∶ 240 µm, counting frame: 100 µm×100 µm; counting grid: 300 µm×300 µm), [25], [26]. We also determined the frequency of newborn neurons, by using confocal microscopy (focal plane of 1 µm) to quantify cells double-stained for NeuN and BrdU staining. On average, we analyzed 100 BrdU-positive cells in each animal (3 animals/group).

### Statistical analysis

We expressed data as means±SEM. Statistical tests are described in figure legends. We performed statistical analysis using GraphPad Prism (version 5.0c, USA). We used the Bonferroni post-hoc test when the one-way ANOVA (analysis of variance), two-way ANOVA or two-way repeated measures (RM) ANOVA revealed significant differences.

## Results

### α-syn mice overexpress WT α-synuclein in the olfactory bulb

**Figure 4:**
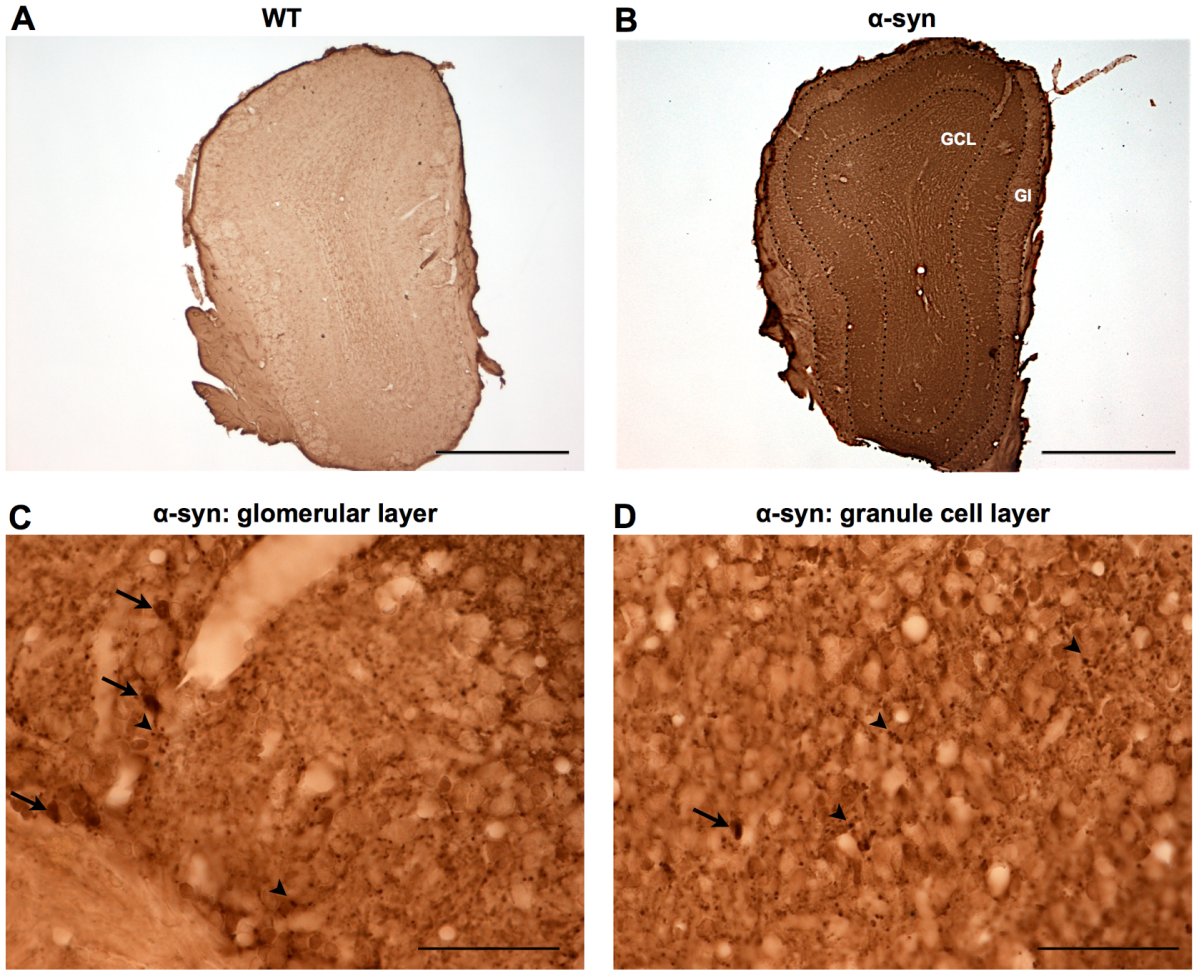
Overexpression of α-synuclein in the olfactory bulb of the α-syn transgenic mice. Immunostaining of human wild-type α-synuclein in OB of **A.** WT mice and **B-D.** α-syn mice aged 12 months. **A-B.** Scale bars: 500 µm. **C-D.** High magnification of **C.** the glomerular layer (Gl) and **D.** the granule cell layer (GCL). Scale bars: 50 µm. α-Syn mice exhibit high expression of human α-synuclein in the different layers of the OB. α-Synuclein immunoreactivity indicates large profiles (arrows) as well as numerous small α-synuclein immunoreactive puncta (arrow heads).

We observed significantly higher levels of α-synuclein immunoreactivity in the OB in the transgenic mice (Figure 4A-B). In different layers of the OB, including the glomerular and granule cell layers, we observed few large (approximately 4 µm diameter) and several smaller α-synuclein immunoreactive profiles (Figure 4C-D).

### Olfactory deficits in the mouse model of PD

#### α-Syn mice show odor detection impairment

In the odor detection test, control mice detected an odor at the dilution 1∶10^6^, whereas α-syn mice needed a higher concentration (dilution 1∶10^4^) indicating that their ability to detect odor is significantly impaired. Neither WT nor α-syn mice were able to detect the lowest odor concentration (1∶10^8^) (Figure 2B).

#### α-Syn mice have short-term olfactory memory deficit

In the short-term olfactory memory test, we found that control mice spent less time sniffing the novel odor during the 2^nd^ exposure, for all the three ITI tested, indicating that they remembered the odor (Figure 2D). α-Syn mice also remembered the odor after ITIs of 60 s and 90 s, but after an ITI of 120 s, they behaved as if they could not remember that they had been exposed to the odor before.

#### α-Syn mice have impaired odor discrimination

In the social odor discrimination test, we found that the α-syn mice were impaired compared to control mice at both odor intensity levels (Figure 2G). The non-social odor discrimination test yielded similar results (Figure 2H). By contrast, control and α-syn mice spent similar time exploring the novel item, meaning that α-syn mice using visual input can discriminate between novel and familiar items (Figure 2H). We also examined if mice prefer either of the two non-social odors we used (Figure 3A). This was not the case, as when they were exposed to lime and lemon odors, both WT and α-syn mice spent equal time sniffing the odors (Figure 3B).

#### α-Syn mice show similar activity and motor ability compared to WT mice

In the open field, α-syn mice traveled a similar distance to control mice (Figure 3C). Likewise, α-syn mice spent similar time compared to control mice in the different areas of the open field (Figure 3D). Finally, control and α-syn mice spent a similar time on the rotarod (Figure 3E). Thus the α-syn mice did not exhibit any signs of anxiety or deficits in locomotor activity and motor function, which could have interfered with the interpretation of odor tests.

### Olfactory deficits are age-dependent

To better characterize the olfactory deficits in this transgenic model and to determine if they are progressive with age, we assessed these deficits in animals aged 3, 11 and 18 months.

#### α-Syn mice exhibit an age-dependent odor detection impairment

**Figure 5:**
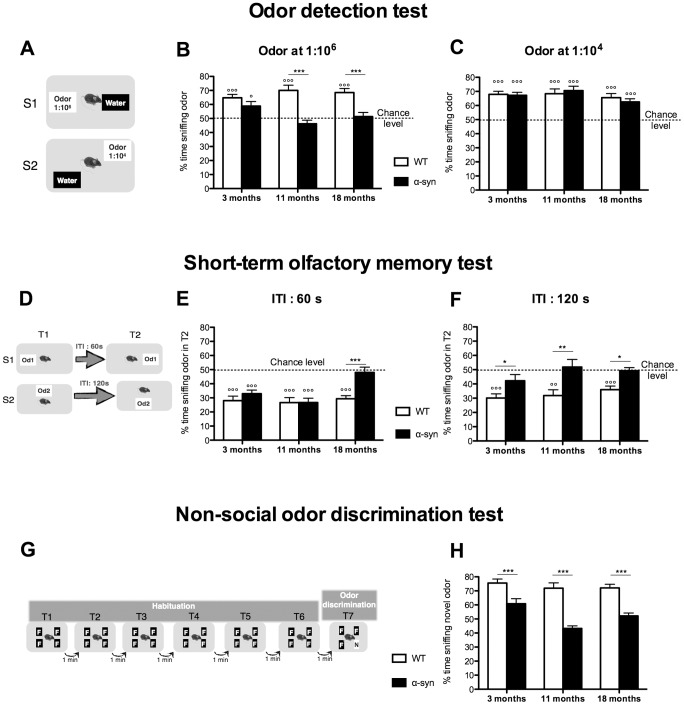
Olfactory deficits are age-dependent. A-C: Odor detection test. Description of the protocol consisting of 2 sessions (S). **B.** Percentage of time spent sniffing the odor at the concentration of 1∶10^6^ (session 1). WT mice aged 3, 11 and 18 months could detect the odor and the percentage of time sniffing the odor was significantly different from the chance level (50%) (°°°p<0.001). On the contrary, α-syn mice are progressively impaired in detecting the odor. Whereas at 3 months transgenic mice spent more time sniffing the odor compared to the chance level (p<0.05), from 11 months of age their scores no longer differed from the chance level (p>0.05) and the percentage of time spent by α-syn mice to sniff the odor is significantly different from WT mice (two way ANOVA). Statistics: One-sample t-tests to compare each value to chance level (50%) (°p<0.05, °°°p<0.001). Two-way ANOVA: age, p = 0.49, F(2,70) = 0.71; genotype, p<0.0001, F(1,70) = 40.21; age×genotype, p = 0.016, F(2,70) = 4.42; Bonferroni post-hoc (***p<0.001). **C.** Percentage of time spent sniffing the odor at the concentration of 1∶10^4^ (session 2). Both WT and α-syn mice aged 3, 11 and 18 months can detect the odor at the concentration of 1∶10^4^ and their percentage of time sniffing the odor is significantly different from the chance level (°°°p<0.001). Moreover, there is no significant difference between the genotypes (two-way ANOVA p>0.05). Statistics: One-sample t-tests to compare each value to chance level (50%) (°°°p<0.001). Two-way ANOVA: age, p = 0.12, F(2,70) = 2.15; genotype, p = 0.83, F(1,70) = 0.045; age×genotype, p = 0.64, F(2,70) = 0.45. **D-F: Short-term olfactory memory test. D.** Description of the protocol consisting of 2 sessions (S). **E.** Session 1 with an inter-trial interval of 60 s. Percentage of time spent sniffing the odor during T2 (trial 2) compared to the total time spent sniffing during both trials. All groups of WT mice aged 3, 11 and 18 months as well as α-syn mice aged 3 and 11 months remember the odor during the 2^nd^ exposure and their percentage of time sniffing the odor during T2 is significantly different from the chance level (50%) (°°°p<0.001). However, from 18 months of age, α-syn mice are impaired in remembering the odor during the 2^nd^ exposure (one-sample t-test, p>0.05) and the percentage of time spent sniffing the odor during T2 is significantly higher compared to 18 month-old WT mice (two-way ANOVA, p<0.001). Statistics: One-sample t-test to compare each value to chance level (50%) (°°°p<0.001). Two-way ANOVA: age, p = 0.0010, F(2,70) = 7.63; genotype, p = 0.0032, F(1,70) = 9.32, age×genotype, p = 0.011, F(2,70) = 4.78; Bonferroni post-hoc (***p<0.001). **F.** Session 2 with an inter-trial interval of 120 s. Percentage of time spent sniffing the odor during T2 compared to the total time spent sniffing during both trials. All groups of WT mice aged 3, 11 and 18 months remember the odor during the 2^nd^ exposure and their percentage of time spent sniffing the odor during T2 is significantly different from the chance level (one-sample t-tests, °°°p<0.001). On the contrary, α-syn mice aged 3, 11 and 18 months, are all impaired in remembering the odor during the 2^nd^ exposure (one-sample t-tests, p>0.05) and the percentage of time spent sniffing the odor during T2 is significantly higher compared to WT mice of the same age (two way ANOVA, *p<0.05, **p<0.01). Statistics: One-sample t-tests to compare each value to chance level (50%) (°°p<0.01, °°°p<0.001). Two-way ANOVA: age, p = 0.13, F(2,70) = 2.12; genotype, p<0.0001, F(1,70) = 26.86; age×genotype, p = 0.53, F(2,70) = 0.64; Bonferroni post-hoc (*p<0.05, **p<0.01). **G-H: Odor discrimination test. G.** Description of the protocol consisting of 6 habituation trials and one odor discrimination trial. **H.** Percentage of time spent sniffing the novel odor. At 3, 11 and 18 months, α-syn mice spend significantly less time compared to age-matched control mice to sniff the novel odor suggesting that they are impaired in their ability to discriminate the novel odor (two-way ANOVA, ***p<0.001). Statistics: Two-way ANOVA: age, p = 0.0028, F(2,70) = 6.42; genotype, p<0.0001, F(1,70) = 77.78; age×genotype, p = 0.077, F(2,70) = 2.66; Bonferroni post-hoc (***p<0.001). For all tests, N = 14 for group aged 3 and 18 months; N = 10 for group aged 11 months.

In the odor detection test (Figure 5A) using the dilution 1∶10^6^, α-syn mice were impaired at 11 and 18 months, whereas at 3 months the performances of WT and α-syn mice were similar (Figure 5B). Using the lower dilution (1∶10^4^), for the 3 ages studied (3, 11 and 18 months) both WT and α-syn mice were able to detect the odor (Figure 5C). Thus, α-syn mice became progressively impaired at detecting the odor at the concentration 1∶10^6^ with aging.

#### With age, α-syn mice show a progressive short-term olfactory memory deficit

In the short-term olfactory memory test (Figure 5D) using the shorter ITI (60 s), α-syn mice aged 3 and 11 months remembered the odor and performed similarly to WT mice whereas they became impaired when they reached 18 months of age (Figure 5E). When mice were tested with the longer ITI (120 s), all α-syn mice failed to remember the odor whatever their age (3, 11 or 18 months) (Figure 5F). Taken together, the short-term olfactory memory deficit of α-syn mice progressively increases with age.

#### α-Syn mice show an age-dependent non-social odor discrimination deficit

In the non-social odor discrimination test (Figure 5G), α-syn mice aged 3, 11 and 18 months were impaired compared to WT mice. Interestingly, two-way ANOVA analysis indicated that age had a significant effect on the short-term olfactory memory performance (Figure 5H).

### Rasagiline improves olfaction in α-syn mice

We evaluated whether 4–8-week rasagiline treatment ameliorates the olfactory deficit exhibited in the α-syn mouse model of PD aged 10–11 months.

#### Rasagiline improves odor detection in α-syn mice

**Figure 6:**
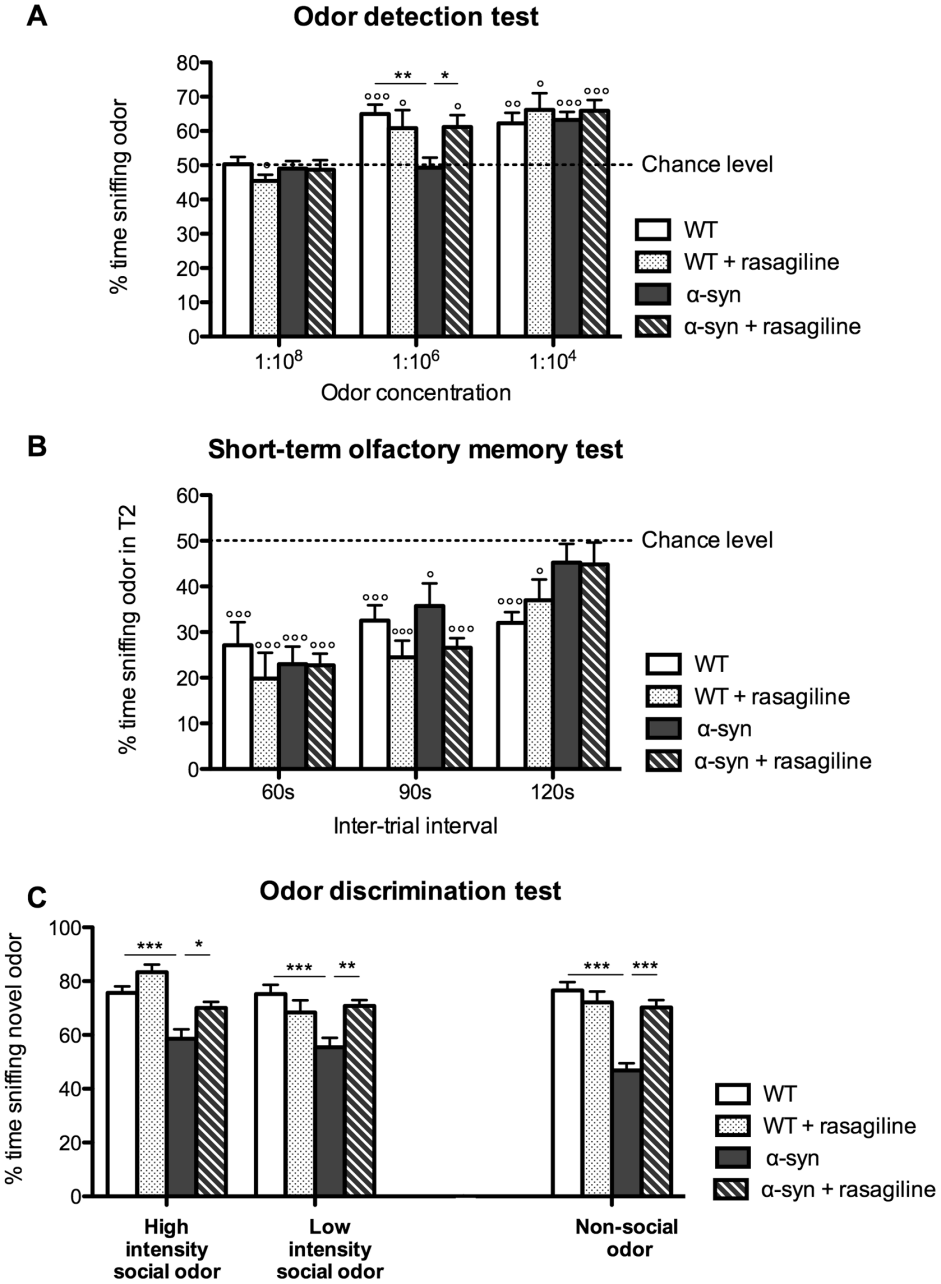
Rasagiline improved some aspects of olfaction in α-syn mice. A. Effect of rasagiline on odor detection deficit in α-syn mice. Rasagiline rescued the odor detection deficit in α-syn mice. At a concentration of 1∶10^6^, non-treated α-syn mice do not detect the odor and the percentage of time spent sniffing the odor was close to chance level, whereas rasagiline treated mice were significantly higher than the chance level. Moreover, rasagiline treated mice spent a similar time sniffing the odor compared to control mice. N = 9–10 for each group aged 10–11 months. Statistics: One-sample t-test to compare each value to chance level (50%), (°p<0.05, °°p<0.01, °°°p<0.001). Two-way RM ANOVA: odor concentration, p<0.0001, F(2,66) = 29; group, p = 0.19, F(3,66) = 1.67; odor concentration×group, p = 0.06, F(6,66) = 2.12; Bonferroni post-hoc (*p<0.05, **p<0.001). **B. Effect of rasagiline on short-term olfactory memory impairment in α-syn mice.** For the 120 s-ITI, percentage of time spent sniffing the odor in T2 was not different from chance level for both α-syn mice groups, treated or not treated with rasagiline. Rasagiline did not improve the short-term olfactory memory in α-syn mice. N =  9–10 for each group aged 10–11 months. Statistics: One-sample t-test compare to chance level (50%), (°p<0.05 and °°°p<0.001). Two-way RM ANOVA: ITI, p<0.0001, F(2,68) = 15.65; group, p = 0.13, F(3,68) = 2.04; ITI×group, p = 0.23, F(6,68) = 1.39; Bonferroni post-hoc. **C. Effect of rasagiline on odor discrimination deficit in α-syn mice.** Percentage of time spent sniffing the novel odor of α-syn mice was increased by rasagiline treatment for both intensities of the social odor as well as for the non-social odor. α-Syn mice treated with rasagiline were similar to control mice (p>0.05). Rasagiline rescued the odor discrimination deficit of α-syn mice. N =  18–21 for each group aged 10–11 months. Statistics for social odor discrimination: Two-way RM ANOVA, odor intensity, p = 0.032, F(1,74) = 4.78; group, p<0.0001, F(3,74) = 13.3; odor intensity×group, p = 0.034, F(3,74) = 3.04; Bonferroni post-hoc (*p<0.05, **p<0.01, ***p<0.001). Statistics for non-social odor discrimination: one-way ANOVA, p<0.001, F(3,73) = 18.16; Bonferroni post-hoc (***p<0.001).

We found that rasagiline treatment normalizes the ability of α-syn mice to detect odors at a concentration (1∶10^6^) when they otherwise are impaired compared to control mice. Whereas the untreated α-syn mice spent a short time (close to chance level) sniffing the novel odor, the time the rasagiline-treated α-syn mice spent sniffing the odor was significantly greater than chance level. Moreover, the α-syn mice treated with rasagiline spent a similar amount of time sniffing a novel odor as the control mice. Thus, rasagiline restores the odor detection ability in α-syn mice to the level of control mice (Figure 6A).

#### Rasagiline does not ameliorate the short-term olfactory memory deficit in α-syn mice

We found that α-syn mice were impaired in their short-term olfactory memory when exposed to a test involving a 120 s ITI. The α-syn mice treated with rasagiline did not exhibit any improvement (Figure 6B).

#### Rasagiline improves the odor discrimination ability of α-syn mice

Rasagiline rescued the social or non-social odor discrimination deficits observed in α-syn mice. Thus, for both odor intensities examined in the social odor discrimination test, α-syn mice treated with rasagiline spent a similar time sniffing the novel odor as control mice and significantly more time sniffing than untreated α-syn mice (Figure 6C). Similarly, α-syn mice were impaired in the non-social odor discrimination test. We found that the rasagiline-treated α-syn mice significantly improved their ability to discriminate non-social odors. Thus, they spent significantly more time sniffing the novel odor compared to non-treated α-syn mice (Figure 6C) and behaved like normal control mice.

### Olfactory bulb neurogenesis is not involved in olfactory deficits in α-syn mice and in the beneficial effect of rasagiline on the deficits

**Figure 7:**
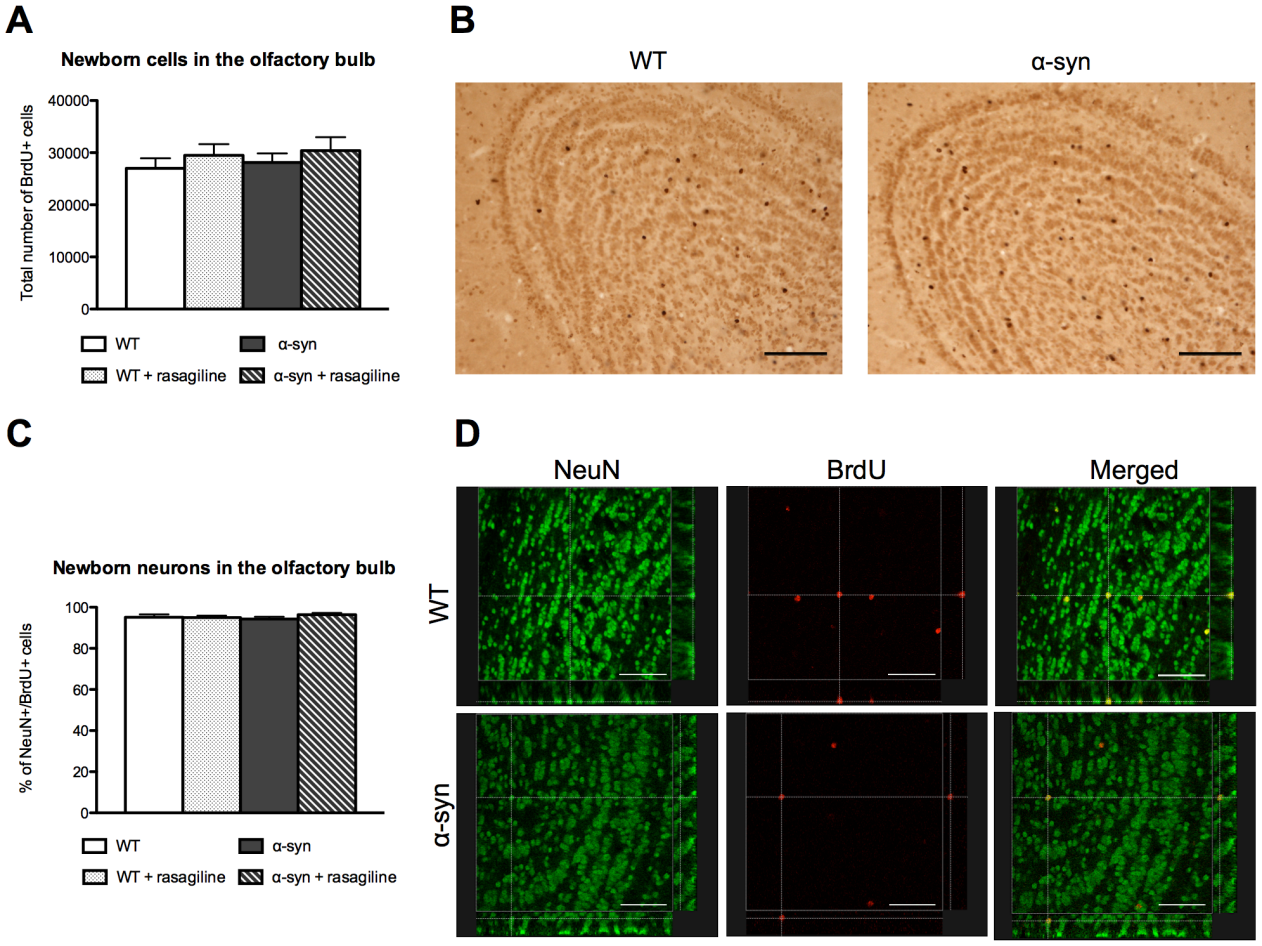
Neurogenesis changes are not involved in the olfactory deficit of α-syn mice and rasagiline-induced improvement. **A.** Quantification of newborn cells in the granule cell layer of the OB. Total number of BrdU positive cells was assessed every sixth section by stereology (counting frame 100 µm×100 µm; counting grid: 300 µm×300 µm). No difference between control and α-syn mice as well as no effect of rasagiline treatment was observed. N = 4-6 for each group aged 12 months. Statistic: one-way ANOVA, p = 0.66, F(3,14) = 0.54. **B.** BrdU staining in the olfactory bulb of WT and α-syn mice. Scale bars: 100 µm. **C. **Quantification of newborn neurons in the granule cell layer of the OB. The proportion of BrdU positive cells, which are also NeuN positive, was assessed by confocal microscopy. No difference between control and α-syn mice as well as no effect of rasagiline treatment was observed. On average, we analyzed 100 BrdU-positive cells in each animal, N = 3 mice in each group aged 12 months. Statistic: one-way ANOVA, p = 0.61, F(3,8) = 0.65. **D.** NeuN (green) and BrdU (red) double staining in the OB. Examples of NeuN-positive/BrdU positive-cells observed in WT and α-syn mice. Scale bars: 55.5 µm.

The number of newborn cells (BrdU-positive cells) in the granule cell layer did not differ between control and α-syn mice, regardless of whether they had been treated with rasagiline or not (Figure 7A-B). Likewise, the percentage of newborn neurons (NeuN-positive/BrdU-positive cells) in the OB granule cell layer was similar in control and α-syn mice, with or without rasagiline treatment (Figure 7C-D).

## Discussion

The F28 transgenic mouse overexpressing human wild-type α-synuclein displays age-dependent olfactory impairments that are manifest as deficits in odor detection, discrimination and short-term memory. Control behavioral tests confirmed that these deficits are specifically due to alterations in olfaction. We found no changes in OB neurogenesis that could explain the olfactory deficits. Importantly, rasagiline improved the ability of α-syn mice to detect and discriminate odors, whereas olfactory short-term memory was unchanged.

### Olfactory deficits in PD mouse models

We found that α-syn mice are impaired in their ability to detect an odor compared to control mice. They also exhibited impaired recollection of an odor after an inter-trial interval of 120 s. Interestingly, odor detection and short-term olfactory deficits have been described in mice after disruption of OB neurogenesis by treatment with the mitosis inhibitor AraC [27], suggesting a role for newborn olfactory interneurons in these functions. Even though OB neurogenesis was not impaired in the α-syn mice, it is interesting to note that these olfactory functions require olfactory interneurons.

We demonstrated impaired discrimination of social and non-social odors in α-syn mice. Mice exhibited no preference for either lime or lemon odors, indicating that the greater time spent sniffing the lime ("novel odor") was due it being perceived as novel. The discrimination deficit was specific to olfaction because when using visual input α-syn mice were able to discriminate a novel item from familiar ones. Our results are consistent with odor discrimination deficits previously described in a different α-syn mouse model overexpressing human wild-type α-syn under the Thy1 promoter [23].

Although the OB plays a crucial role in odor detection, odor discrimination and short-term olfactory memory, others brain structures, such as the olfactory cortex, could also be involved in olfactory deficits observed in our model. The piriform cortex, for example, is involved in the identification, categorization and discrimination of olfactory stimuli [28]. In the same way, the olfactory tubercle contributes to the odor perception, odor discrimination and higher–order olfactory functions [29]. Moreover, these structures are also affected in PD patients, exhibiting alpha-synucleinopathy [6] and could play a role in the hyposmia related to PD.

Interestingly, in this transgenic model, the deficits to detect, discriminate and to remember an odor during a short time interval are age-dependent, which emphasizes the relevance of this model for the neurodegenerative Parkinson's disease.

### Specific olfaction deficits relevant to PD

Our behavioral studies show clearly that α-syn mice were actually capable of performing each test per se, and that the impairments they exhibited were specifically due to reduced olfactory functions. Thus, although α-syn mice could not detect an odor at a concentration of 1∶10^6^, they were able to detect the same odor at a concentration of 1∶10^4^. In the test of olfactory memory, they failed to remember an odor presented 120 s earlier, but were successful in doing so when the inter-trial interval was as short as 60 s or 90 s. Moreover, while they were impaired in discriminating a novel odor, α-syn overexpressing mice were normal when it came to discriminating novel objects. Finally, in the test for motor function and anxiety, the α-syn mice did not differ from control mice, indicating that changes in these behavioral parameters were unlikely to be involved in the observed olfactory impairments.

The olfactory deficits we observed in the mouse PD model are consistent with clinical observations of impairments in the abilities to detect, discriminate and identify odors in idiopathic PD [30]–[34]. Therefore, our animal model is relevant to the clinical setting.

### Do changes in neurogenesis or neuronal activity cause olfactory deficits?

We hypothesized that the olfactory deficits were due to alterations in OB neurogenesis. Reduced OB neurogenesis has previously been associated with deficits in odor detection and short-term olfactory memory [27]. Contrary to previous studies showing reduced OB neurogenesis in transgenic mice overexpressing either human wild-type or mutant α-synuclein [8], [35], we did not detect any reduction of newborn cells or neurons in the OB. Therefore, the olfactory deficits we observed are not likely to be due to changes in OB neurogenesis. Two earlier studies used mice expressing the transgene under a different promoter (PDGF whereas we used mouse α-synuclein) [8], [35], which may lead to a different pattern and level of α-syn overexpression, explaining the differences in our results.

An alternate explanation for the olfactory impairment is that overexpression of α-syn in the OB directly affects local neuronal activity. We found that α-syn is highly overexpressed in the OB of our transgenic model, in particular in the glomerular layer including dopaminergic periglomerular interneurons, in the mitral cell layer and the granule cell layer. Moreover, these cells and layers of the OB are clearly involved in the olfactory functions that we found to be impaired in the α-syn mice [27], [36].

### Rasagiline reverses certain olfactory deficits

We found that rasagiline treatment improved olfaction of α-syn mice and rescued odor detection and odor discrimination deficits. Rasagiline did not, however, ameliorate short-term olfactory deficits. Since we did not observe any reduction of neurogenesis in α-syn mice, nor any positive effect of rasagiline on neurogenesis, it is highly unlikely that the improved olfaction following rasagiline treatment is related to enhanced neurogenesis.

The rasagiline dose used (3 mg/kg) has previously been found to be efficacious in models of cerebral ischemia [37], vitamin deficiency [38] and PD [16]. We chose a long-term treatment (4–8 weeks) because we were not only interested in the MAO-B inhibitory activity of rasagiline [39], but also in its potential neuroprotective effects [16], [40].

The rasagiline metabolite aminoindan is reported to be neuroprotective in several models of neuronal damage. This effect appears to be independent of MAO-B inhibition [41]. One potential mode of action of rasagiline is the stabilization of the mitochondrial membrane potential [42]. Interestingly, α-synuclein interacts directly with mitochondrial membranes [43], inhibits complex I [44], and thereby reduces the mitochondrial membrane potential [45]. Moreover, mitochondria in transgenic mice overexpressing mutant α-syn have been reported to display abnormal structure and function [46], [47]. Therefore it is plausible that overexpression of α-syn directly affects mitochondria and thereby impairs neuronal function, and that rasagiline could potentially mitigate these effects.

Another option is that rasagiline, which is known to inhibit MAO-B, could improve endogenous dopamine concentrations and transmission [39]. The rasagiline dose (3 mg/kg/day) can reduce the residual MAO-B activity in the brain from 2% to 0.08% compared to untreated controls [38], [48]. Knowing that MAO-B is expressed in the olfactory bulb [49], [50], it is likely that rasagiline therapy could affect dopamine transmission in the olfactory bulb especially between interneurons and olfactory receptor neurons or mitral/tufted cells in the glomerular layer. These cells are involved in odor detection and discrimination, functions, which are both improved by rasagiline treatment. Interestingly, dopamine receptor (D1 or D2) agonists or antagonists, affect odor discrimination learning as well as odor detection threshold [51]–[53]. In the same vein, transgenic mice lacking either dopamine transporters or D2 dopamine receptors exhibit odor discrimination impairment [22] suggesting that D2 dopamine receptor activation is important for odor discrimination. Therefore, the MAO-B inhibitory activity of rasagiline might underlie the beneficial effects on odor discrimination and detection.

In conclusion, our study shows a robust positive effect of rasagiline treatment on olfactory deficits in a transgenic mouse model of PD. The underlying mechanisms require further elucidation. Meanwhile it would be valuable to systematically examine if rasagiline improves olfaction in PD patients.

## Supporting Information

Figure S1 **Plastic cartridge and wood block used in the olfactory tests. A.** The cartridge is a plastic tube (eppendorf), open at the two extremities, filled with a piece of compress. The compress is not accessible to the mice. During olfactory tests, odor solutions are prepared daily and we apply 400 µl of the solution (200 µl each side) to the compress. As both ends of the tubes are open, the odor can easily diffuse during the tests. **B.** The wood block is approximately 3 cm^3^. During the impregnation time, wood blocks will get mouse odors mainly coming from mouse' body fluids.
(TIF)
