# Supplementary figures and images for: Rasagiline Ameliorates Olfactory Deficits in an Alpha-Synuclein Mouse Model of Parkinson's Disease
Source: PLoS One. 2013 Apr 3;8(4):e60691. doi: 10.1371/journal.pone.0060691 (PMC3616111; doi:10.1371/journal.pone.0060691)

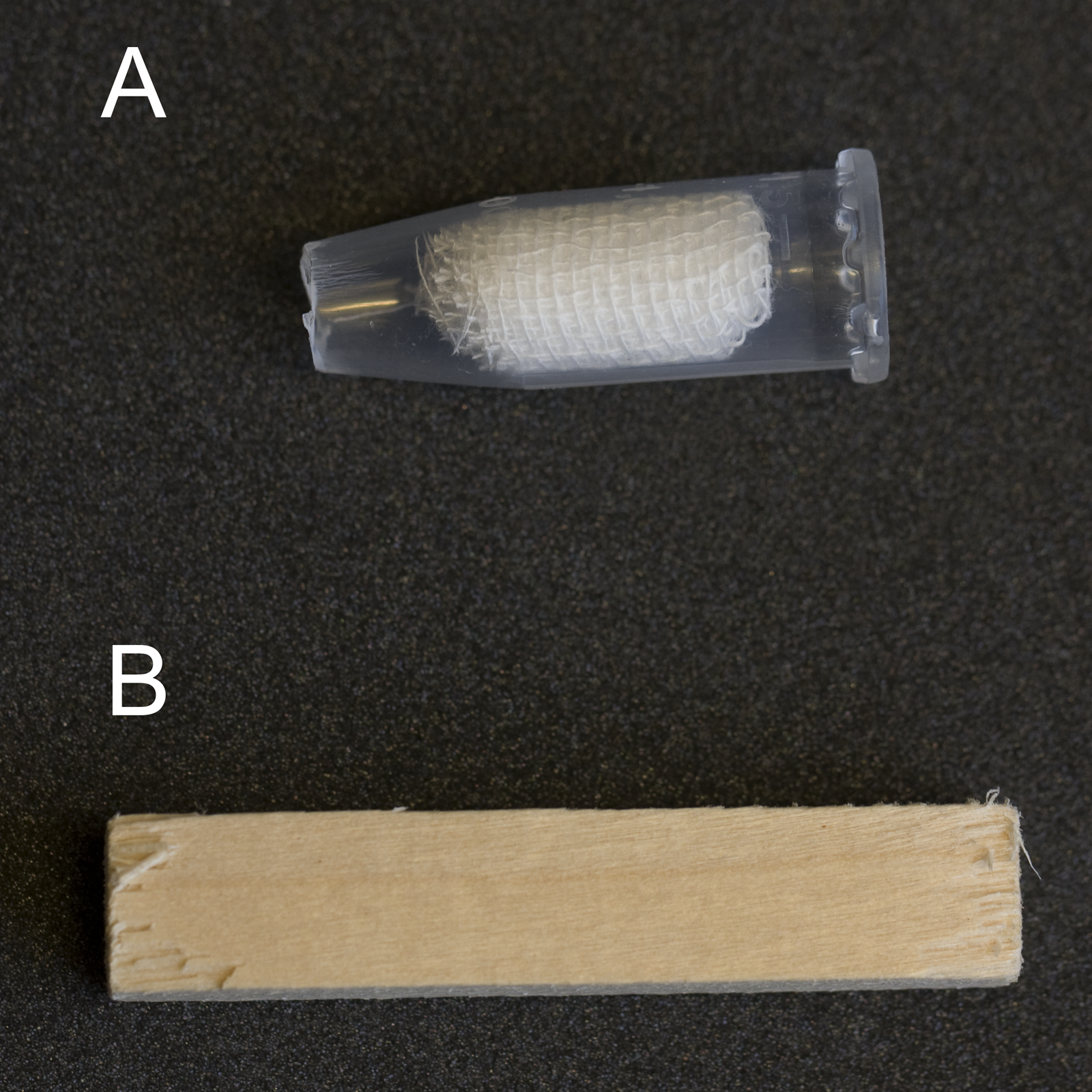

Supplement: Figure S1 — Plastic cartridge and wood block used in the olfactory tests. A. The cartridge is a plastic tube (eppendorf), open at the two extremities, filled with a piece of compress. The compress is not accessible to the mice. During olfactory tests, odor solutions are prepared daily and we apply 400 µl of the solution (200 µl each side) to the compress. As both ends of the tubes are open, the odor can easily diffuse during the tests. B. The wood block is approximately 3 cm3. During the impregnation time, wood blocks will get mouse odors mainly coming from mouse' body fluids. (TIF) [file pone.0060691.s001.tif]
